# Administration of the American Board of Anesthesiology’s virtual APPLIED Examination: successes, challenges, and lessons learned

**DOI:** 10.1186/s12909-024-05694-7

**Published:** 2024-07-11

**Authors:** Mark T. Keegan, Ann E. Harman, Thomas M. McLoughlin, Alex Macario, Stacie G. Deiner, Robert R. Gaiser, David O. Warner, Santhanam Suresh, Huaping Sun

**Affiliations:** 1 Department of Anesthesiology and Perioperative Medicine, Mayo Clinic, Rochester, MN 55905 USA; 2 The American Board of Anesthesiology, 4200 Six Forks Rd, Suite 1100, Raleigh, NC 27609 USA; 3 Department of Anesthesiology, Lehigh Valley Health Network, Allentown, PA 18103 USA; 4 Department of Anesthesiology, Perioperative and Pain Medicine, Stanford University, Stanford, CA 94305 USA; 5 Department of Anesthesiology, Dartmouth Hitchcock Medical Center, Lebanon, NH 03756 USA; 6 Department of Anesthesiology, Yale School of Medicine, New Haven, CT 06510 USA; 7 Department of Anesthesiology, Northwestern University Feinberg School of Medicine, Chicago, IL 60611 USA

**Keywords:** Virtual APPLIED Examination, Virtual Objective Structured Clinical Examination, Virtual Standardized Oral Examination, Virtual candidate experiences, Virtual examiner experiences

## Abstract

In response to the COVID-19 pandemic, the American Board of Anesthesiology transitioned from in-person to virtual administration of its APPLIED Examination, assessing more than 3000 candidates for certification purposes remotely in 2021. Four hundred examiners were involved in delivering and scoring Standardized Oral Examinations (SOEs) and Objective Structured Clinical Examinations (OSCEs). More than 80% of candidates started their exams on time and stayed connected throughout the exam without any problems. Only 74 (2.5%) SOE and 45 (1.5%) OSCE candidates required rescheduling due to technical difficulties. Of those who experienced “significant issues”, concerns with OSCE technical stations (interpretation of monitors and interpretation of echocardiograms) were reported most frequently (6% of candidates). In contrast, 23% of examiners “sometimes” lost connectivity during their multiple exam sessions, on a continuum from minor inconvenience to inability to continue. 84% of SOE candidates and 89% of OSCE candidates described “smooth” interactions with examiners and standardized patients/standardized clinicians, respectively. However, only 71% of SOE candidates and 75% of OSCE candidates considered themselves to be able to demonstrate their knowledge and skills without obstacles. When compared with their in-person experiences, approximately 40% of SOE examiners considered virtual evaluation to be more difficult than in-person evaluation and believed the remote format negatively affected their development as an examiner. The virtual format was considered to be less secure by 56% and 40% of SOE and OSCE examiners, respectively. The retirement of exam materials used virtually due to concern for compromise had implications for subsequent exam development. The return to in-person exams in 2022 was prompted by multiple factors, especially concerns regarding standardization and security. The technology is not yet perfect, especially for testing in-person communication skills and displaying dynamic exam materials. Nevertheless, the American Board of Anesthesiology’s experience demonstrated the feasibility of conducting large-scale, high-stakes oral and performance exams in a virtual format and highlighted the adaptability and dedication of candidates, examiners, and administering board staff.

**Supplementary Information:**

The online version contains supplementary material available at 10.1186/s12909-024-05694-7.

## Introduction

The third and final step in the American Board of Anesthesiology’s (ABA; Raleigh, NC) staged examination process for initial certification [1–4] is the APPLIED Examination, which consists of two components, a Standardized Oral Examination (SOE) [3] and an Objective Structured Clinical Examination (OSCE) [4–6]. The SOE includes two 35-minute sessions, during which candidates answer examiners’ guided questions about the scientific rationale underlying clinical management decisions [3]. The OSCE includes five Communication & Professionalism stations based on clinical scenarios, in which candidates interact with actors playing standardized patients or standardized clinicians, and two Technical Skills stations, during which candidates are asked to interpret monitors, echocardiograms, or to apply ultrasonography [4]. The APPLIED Examination is usually administered at a dedicated assessment center in Raleigh, North Carolina to approximately 2000 candidates per year. Candidates must pass both components to pass the APPLIED Examination; they may retake any failed component(s). In 2020, disruption associated with the COVID-19 pandemic led to the cancellation of the on-site examinations, requiring the development and implementation of a remote, internet-based form of both components of the examination – the ABA Virtual APPLIED Examination (VAE).

The processes used to develop the ABA VAE and deliver it using the Zoom platform (Zoom Video Communications, San Jose, CA) have been described in detail [7]. We have also reported the psychometric performances of the VAE by comparing the virtual formats of the SOE and the OSCE with their in-person equivalents [8, 9]. Candidate performance and examiner grading severity were comparable between the in-person and virtual formats for both the SOE and the OSCE, supporting the reliability and validity of the virtual examinations, although OSCE scenarios delivered virtually were more difficult than those delivered in-person. In this paper, we detail the challenges, successes, and failures of operational logistics and administration of this high-stakes, career-defining physician assessment during the pandemic. In addition to a narrative review, we provide survey-generated performance data for our communication strategies, technology infrastructure, and staffing models. Further, we present candidate and examiner perceptions of the examination infrastructure and their exam experiences. The practicalities of virtually delivering the ABA’s certifying examination during the pandemic documented in this paper will provide insight for other assessment organizations that may need to deploy a large-scale, high-stakes, virtual performance exam in the future.

## Candidates and examiners

Candidates had completed Accreditation Council for Graduate Medical Education (Chicago, IL)-accredited anesthesiology residency training and had passed the ABA BASIC [1] and ADVANCED [2] examinations. The majority of VAE candidates completed their residency in 2019 or 2020. Those who graduated in 2019 and had their APPLIED Examinations canceled in 2020 were examined during VAE pilot testing in December 2020 or during one of nine weeks between February and April 2021 (VAE Window 1). Residency graduates from 2020 were examined between July and November 2021 (VAE Window 2). Examiners were volunteer ABA board-certified anesthesiologists who were clinically active and participating in the Maintenance of Certification in Anesthesiology program [10, 11]. Examiners for in-person and virtual SOEs and OSCEs were drawn from the same examiner pool.

## Surveys

Evaluation of VAE candidate and examiner experiences was planned prior to VAE implementation; survey results were described in the appropriate sections of this narrative review. The surveys were determined by the WCG Institutional Review Board (Puyallup, WA) to be exempt from review.

All candidates and examiners who participated in either or both components of the VAE were invited by email to respond to anonymous online surveys, using SurveyMonkey (San Mateo, CA). Candidates and examiners who had both in-person and virtual experiences were invited to complete *separate* online surveys that specifically queried how their virtual SOE or OSCE experiences compared with their previous in-person examinations, using QuestionPro (Beaverton, OR). Most virtual candidates were first-time takers of the APPLIED Examination and so could not compare in-person and virtual experiences, but VAE Window 1 included some candidates who had previously failed the in-person SOE, or the in-person OSCE, or both. Candidates were invited to their survey(s) within days of examination administration and could respond until exam results were released. Examiners received survey invitations after concluding their assigned examination weeks. Full or partial completion of the survey was taken as an indication of consent for participation.

Both candidates and examiners were asked about their perception of communications from the ABA before the examinations, their experiences regarding the technology infrastructure required to conduct the exams, and their reflections on the process of taking or conducting/scoring the exams. In addition to questions on the Likert scales, respondents could provide comments to multiple open-ended questions. The candidate and examiner comparison surveys focused on how the virtual delivery of the SOE or OSCE affected their preparation effort, perceived professionalism level of others, interaction between candidates and examiners, and how the virtual format affected their ability to demonstrate or evaluate the qualities that the SOE or OSCE is designed to assess.

Of the 3059 candidates who took the VAE, 1452 (47%) responded to the VAE survey. The vast majority of these candidates (95%) had taken both virtual SOE and virtual OSCE, almost all on the same day. Of the 228 candidates who had previously failed an in-person SOE, 113 (50%) responded. Of the 56 candidates who had previously failed an in-person OSCE, 28 (50%) responded. Among 317 examiners who had conducted both the in-person and virtual SOEs and were invited to the SOE examiner comparison survey, 201 (63%) completed the survey. Among 254 examiners who scored both the in-person and virtual OSCEs and were invited to the OSCE examiner comparison survey, 170 (67%) completed the survey.

## VAE pilot

Eighty-eight (88) candidates voluntarily participated in a pilot administration of the VAE over two days in December 2020, and all took both the SOE and the OSCE. Fifty-two (52) SOE examiners examined a median of 8 candidates each (range 1–8), and 43 examiners scored a median of 14 OSCE candidate-stations (range 3–42). Those candidates who passed both SOE and OSCE became ABA certified; those who failed one or both components were offered an opportunity to retake the exam in early 2021. As previously described, the pilot went sufficiently well that the full-scale VAE proceeded, but many opportunities for improvement were identified and addressed before the first operational administration in February 2021 [7]. Pilot administration survey results are reported in Supplemental Tables 1 and 2.

Based on survey feedback from the pilot, changes to the SOE process included the provision of a “one minute remaining” warning to examiners and candidates, a virtual transition room for candidates after their interactions with examiners concluded and before they were disconnected from Zoom, and a smoother exam end for examiners (rather than abruptly ending their Zoom session). For the OSCE, modifications were made to improve the on-screen scenario display, to make transitions between stations smoother, and to explain the technical station process more clearly. Lastly, pre-examination information and preparatory materials for operational virtual exam weeks were revised and sent to candidates and examiners sooner than for the pilot.

## VAE logistics

The *operational* administration of the ABA VAE began on Feb. 1, 2021, and continued through Nov. 18, 2021. In this period, 3059 candidates were examined, including 2916 taking both the SOE and OSCE, 95 taking the SOE only (83 for Part 2 in the traditional exam system and 12 for the SOE only in the staged exam system), and 48 taking the OSCE only. The total number of candidates examined in 2021 was 1.7 times the candidate volume examined in a typical year (Table 1). This cleared the backlog of candidates whose exams were postponed because of the pandemic (and who chose to take the exam in 2021), while examining those who became eligible to take their exams in 2021. Four hundred (400) examiners participated in the VAE, with 340 administering and scoring SOEs, 279 scoring OSCEs, and 219 participating in both.

**Table 1:** Virtual APPLIED Exam delivery results and irregularities

Week	w1	w2	w3^*^	w4	w5	w6	w7	w8	w9	w10	w11	w12	w13	w14	w15	w16	w17	Total
**Candidate volume**																		
SOE + OSCE	164	185	123	158	197	152	142	147	170	167	171	187	182	171	184	207	209	2916
SOE/Part 2^†^ only	11	8	6	6	6	5	7	7	7	1	1	0	6	5	4	10	5	95
OSCE only	9	5	8	3	3	4	2	0	1	0	1	1	2	3	1	2	3	48
*Total # of all candidates examined*	*184*	*198*	*137*	*167*	*206*	*161*	*151*	*154*	*178*	*168*	*173*	*188*	*190*	*179*	*189*	*219*	*217*	*3059*
**SOE/Part 2 irregularities**																		
# of candidates rescheduled due to technical issues (including time zone issues)	8	2	2	1	9	5	3	9	3	0	2	1	7	2	9	6	5	74
# of single examiner rooms	13	4	4	0	0	1	0	1	0	5	3	1	1	3	1	0	3	40
# of examiner cancellations (full or partial week)	3	6	3	6	7	4	9	*Not tracked*										38
# of candidate “no-shows”	1	6	2	1	1	2	3	0	2	2	2	0	3	3	1	2	4	35
**OSCE irregularities﻿**																		
# of invalidated OSCE stations	11	8	1	2	3	6	1	3	2	5	3	2	5	3	7	5	3	70
# of candidates rescheduled due to technical issues (including time zone issues)	2	2	3	1	4	2	1	9	3	0	2	2	8	1	0	0	5	45
# of candidate “no-shows”	2	3	2	1	0	2	1	0	0	2	2	0	3	3	1	1	2	25

* A power outage caused 12 SOEs and 10 OSCEs to be rescheduled in week 3

† Part 2 was the counterpart of the SOE in the traditional examination system, which was the initial certification system prior to the staged examination system

During Window 1 (February – April 2021), candidates were examined on 3 or 4 weekdays per week between 7:30 am and 7:30 pm Eastern Time over 6 exam periods per day. For Window 2 (July – November 2021), candidates were examined Monday through Thursday between 8:30 am and 4:30 pm Eastern Time, using the pre-pandemic in-person schedule of 4 periods per day and 4 days per week. The additional periods 5 and 6 scheduled during Window 1 were to accommodate candidates in the Mountain and Pacific time zones and candidates who needed to reschedule on the same day due to technical issues. These periods were under-utilized in Window 1 and were thus not scheduled during Window 2.

Five ABA APPLIED Examination staff engaged in exam scheduling and supporting exam delivery, supplemented by 19 temporary staff hired for the role of exam facilitators. The organization of examination support staff has been described previously [7]. Twenty-eight (28) professional actors played the roles of standardized patients and/or standardized clinicians. Examinations were scheduled across 5 time zones, with 15 virtual SOE and 14 virtual OSCE “rooms” running simultaneously. In addition, we accommodated a small number of candidates in other time zones who were deployed overseas on active-duty military service.

## Irregular events

Contingency plans were in place for many - but not all - eventualities. For example, during Week 3 there was a relatively brief power outage at the assessment center, from where the VAE was coordinated, causing the postponement of 12 SOEs and 10 OSCEs. Early in the administration, a few candidates were examined by a single examiner in one of their SOE sessions, because the other examiner experienced technical difficulties and there was not enough time to bring in a replacement examiner. These candidates were later scored asynchronously by a second examiner based on examination recordings (Table 1). In each case of “irregularity”, ABA Directors discussed the details with APPLIED Exam staff to understand the nature of the deviation from the norm and decided whether an SOE session or OSCE station should be allowed to proceed, invalidated, or rescheduled, with the intention to favor candidates to a reasonable extent. For example, if a technical issue (e.g., audio problems because of poor internet connectivity or suboptimal microphone recording) caused an individual OSCE station to be unscoreable, the candidate would be awarded the highest possible score for that station as long as their six other OSCE stations were scored under “normal” conditions. In situations where communication between a candidate and examiners during an SOE session was disrupted beyond an acceptable threshold, the candidate was given the opportunity to re-test on the same or subsequent day.

## Standards and pass rates

**Fig. 1:**
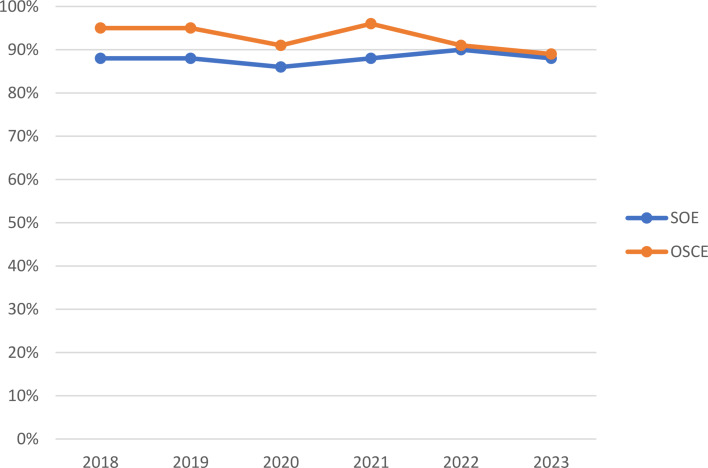
2021 virtual Standardized Oral Examination (SOE) and Objective Structured Clinical Examination (OSCE) pass rates in comparison to those of in-person APPLIED Exams. *Note*: the 2020 pass rates include a one-week March 2020 in-person exam and a December 2020 two-day virtual pilot exam

Scoring the SOE and the OSCE uses the many-facet Rasch model and the techniques have been described previously [3, 4, 12]. We have also described the psychometric performances of the virtual SOE and virtual OSCE [8, 9]. After the first cohort of candidates completed the VAE in February 2021, a standard-setting exercise took place for the virtual OSCE because of its structural change from the in-person OSCE (e.g., the ultrasound station was excluded from the virtual OSCE), and the resulting standard was used throughout 2021. For the virtual SOE, the existing standard was reviewed —no substantial changes other than transitioning to the virtual format warranted a new standard. Maintenance of the existing standard was subsequently validated by the stable performance of the virtual SOE candidates. Operational pass rates for both components of the VAE were similar to those seen in previous and subsequent years for in-person exams (Fig. 1).

## Communications from the ABA

A multi-pronged approach was taken to inform candidates and examiners of the details of the VAE, including live webinars with question-and-answer sessions, websites with downloadable materials including procedure manuals and infographics, SOE- and OSCE-specific briefing videos, email communications using an ABA’s APPLIED Exam-specific e-mail address, and telephone helpline [7]. The timeliness of delivery of materials improved for the operational VAE compared with the pilot administration, and both candidates and examiners found each type of material provided to be useful (Table 2). However, despite the early availability of preparatory materials, 3% of 2021 virtual candidates still perceived that materials and examination information were late and “left them scrambling at the last minute” (compared with 8% in the pilot, Supplemental Table 2). Anecdotally, ABA e-mails blocked by institutional firewalls or directed to spam folders accounted for many of these difficulties, despite a counsel to candidates to add the ABA’s dedicated e-mail address to their safe sender list. The detailed candidate- and examiner-specific procedure manuals were perceived as being the most useful preparatory materials.

**Table 2:** Candidate and examiner perceived utility of the ABA-provided exam materials

How useful did you find each of the exam materials?	*N*	Extremely useful	Very useful	Somewhat useful	Not so useful
*Candidate*					
Candidate Procedures Manual	1360	391 (28.8%)	683 (50.2%)	259 (19.0%)	27 (2.0%)
Live Webinar	1041	251 (24.1%)	425 (40.8%)	298 (28.6%)	67 (6.4%)
Virtual Exam Infographic	1180	287 (24.3%)	563 (47.7%)	292 (24.7%)	38 (3.2%)
SOE Overview Video	1325	409 (30.9%)	607 (45.8%)	274 (20.7%)	35 (2.6%)
OSCE Overview Video	1227	400 (32.6%)	526 (42.9%)	263 (21.4%)	38 (3.1%)
*Examiner*					
Examiner Procedures Manual	500	236 (47.2%)	228 (45.6%)	31 (6.2%)	5 (1.0%)
Live Webinar	437	166 (38.0%)	171 (39.1%)	87 (19.9%)	13 (3.0%)
Pre-Exam Checklist	465	200 (43.0%)	190 (40.9%)	64 (13.8%)	11 (2.4%)
Virtual Exam Infographic	379	127 (33.5%)	163 (43.0%)	73 (19.3%)	16 (4.2%)
SOE Briefing Video	344	129 (37.5%)	155 (45.1%)	57 (16.6%)	3 (0.9%)
OSCE Briefing Video	320	126 (39.4%)	152 (47.5%)	39 (12.2%)	3 (0.9%)

## Technology infrastructure

The Zoom platform was used to administer the VAE; an up-to-date Google Chrome web browser was required to generate the videoconference links and to display exam-related material [7]. Both candidates and examiners were required to conduct a “system check” before the day of the examination. The overwhelming majority of systems passed on the first attempt or after minor adjustments. Fewer than 1% of participants (candidates and examiners) had to change either their computer system or their intended physical location for the exam because of the system check (Table 3).

**Table 3:** Candidate and examiner experiences with technology

	*N*	Response				
*Candidate*						
**Which best describes your experience with the system check?**	1404	**My system passed on the first try**	**I had to make a few adjustments to my system before it passed**	**I had to make substantial adjustments to my system before it passed**	**I had to choose a different location and/or network for the exam**	**Other**
		1318 (93.9%)	65 (4.6%)	4 (0.2%)	3 (0.2%)	14 (1.0%)
**Which of the following best describes your experience with the Zoom platform?**	1386	**Exceeded expectations**	**Met expectations**	**Below expectations**		
		393 (28.4%)	898 (64.8%)	95 (6.9%)		
**What best describes your experience of each of the following as it pertains to both sessions of your Standardized Oral Exam (SOE)?**		**No issues**	**A few issues**	**Significant issues**		
Zoom: Audio	1350	916 (67.9%)	395 (29.3%)	39 (2.9%)		
Zoom: Video	1347	1185 (88.0%)	142 (10.5%)	20 (1.5%)		
Zoom: Connectivity	1346	1123 (83.4%)	196 (14.6%)	27 (2.0%)		
Stem: Display	1349	1200 (89.0%)	115 (8.5%)	34 (2.5%)		
Stem: Prep time	1350	1206 (89.3%)	120 (8.9%)	24 (1.8%)		
Stem: Demonstration time	1319	1186 (89.9%)	110 (8.3%)	23 (1.7%)		
**What best describes your experience of each of the following as it pertains to all 7 scenarios of your Objective Structured Clinical Exam (OSCE)?**		**No issues**	**A few issues**	**Significant issues**		
Zoom: Audio	1228	1060 (86.3%)	156 (12.7%)	12 (1.0%)		
Zoom: Video	1233	1120 (90.8%)	93 (7.5%)	20 (1.6%)		
Zoom: Connectivity	1229	1129 (91.9%)	75 (6.1%)	25 (2.0%)		
Stem: Display	1232	1015 (82.4%)	163 (13.2%)	54 (4.4%)		
Stem: Prep time	1229	1019 (82.9%)	161 (13.1%)	49 (4.0%)		
Standardized Patient Experience	1230	1129 (91.8%)	85 (6.9%)	16 (1.3%)		
Technical Stations	1221	939 (76.9%)	210 (17.2%)	72 (5.9%)		
**How often did your exams start at the scheduled time?**	807	**Always**	**Often**	**Sometimes**	**Never**	
		418 (51.8%)	247 (30.6%)	95 (11.8%)	47 (5.8%)	
**How often did you lose connectivity during the exam?**	1385	**Always**	**Often**	**Sometimes**	**Never**	
		8 (0.6%)	24 (1.7%)	192 (13.9%)	1161 (83.8%)	
**Were you aware of any technical issues on the part of your examiner(s)?**	1385	**Yes**	**No**			
		232 (16.8%)	1153 (83.2%)			
***Examiner***						
**Which best describes your experience with the system check?**	514	**My system passed on the first try**	**I had to make a few adjustments to my system before it passed**	**I had to make substantial adjustments to my system before it passed**	**I had to choose a different location and/or network for the exam**	**Other**
		467 (90.9%)	29 (5.6%)	4 (0.8%)	5 (1.0%)	9 (1.8%)
**Which of the following best describes your experience with the Zoom platform?**	509	**Exceeded expectations**	**Met expectations**	**Below expectations**		
		184 (36.1%)	322 (63.3%)	3 (0.6%)		
**How often did your exams start at the scheduled time?**	291	**Always**	**Often**	**Sometimes**	**Never**	
		136 (46.7%)	118 (40.5%)	29 (10.0%)	8 (2.7%)	
**How often did you lose connectivity during the exam?**	508	**Always**	**Often**	**Sometimes**	**Never**	
		4 (0.8%)	0 (0%)	115 (22.6%)	389 (76.6%)	

The majority of candidates (82%) and examiners (87%) “always” or “often” started their exams at the scheduled time; still, a small percentage of candidates (6%) and examiners (3%) “never” started their exams on time (Table 3). Some delays were related to technical difficulties on the first day of the first examination administration week in February 2021, when a coding problem resulted in failure to generate some Zoom meeting links for examiners. Others occurred intermittently over the course of the VAE administration and were almost entirely related to internet connectivity or participant audiovisual problems.

Internet connectivity was generally reliable — 84% of candidates and 77% of examiners never lost connectivity during the exam. For those who reported “sometimes” losing connectivity during the exam (14% of candidates and 23% of examiners), their loss of connectivity was likely on a continuum ranging from audio and/or video interruption lasting a few seconds to a complete inability to continue the exam (Table 3). For 74 (2.5%) SOE candidates and 45 (1.5%) OSCE candidates, connectivity problems were severe enough that their exams had to be rescheduled (Table 1). A higher percentage of examiners reported connectivity issues, perhaps explained by the fact that examiners had more chances for exam interruption than candidates – typically conducting SOEs for multiple candidates. It is worth noting that 10% of candidates were aware of technical issues on the part of their examiners (each candidate is examined by a total of four SOE examiners). On the other hand, about one-third of candidates and examiners reported that Zoom exceeded their expectations, with examiners (36%) being more likely to be unexpectedly impressed than candidates (28%; Table 3), perhaps reflecting a generational divide in the familiarity with audio-visual interfaces [13].

Candidates’ more granular evaluations revealed that they experienced “significant issues” most frequently at OSCE technical stations (interpretation of monitors and interpretation of echocardiograms) at 6%, followed by OSCE stem display (4%) and OSCE stem preparation time (4%; Table 3). Despite explicit instructions in the candidate procedures manual and the availability of the exam facilitator administering the technical stations to troubleshoot, this small portion of candidates had difficulty navigating the combination of Zoom and internet browser windows in the technical stations. This may have contributed to some reports of insufficient OSCE station preparatory time. As previously mentioned, in circumstances of obvious technical disruptions, OSCE stations were rated in the candidate’s favor.

### Perceptions of the virtual SOE

The vast majority of candidates indicated agreement with statements supporting the professionalism of the examiners (93%) and “smooth” interactions with examiners (84%; Table 4). About 70% of SOE candidates agreed that the virtual SOE allowed them to demonstrate their knowledge and skills without obstacles and that the virtual SOE effectively measured their ability to analyze clinical situations, adapt to changing clinical scenarios, make appropriate clinical judgments, or organize and present information; the remaining 30% of candidates were neutral about, disagreed, or strongly disagreed with these statements. Recognizing that surveys were answered before exam results were released, two-thirds of candidates reported a positive experience of the virtual SOE, approximately 30% were neutral, and 4% reported a negative experience.

**Table 4:** Candidate and examiner experiences of the virtual Standardized Oral Examination (SOE)

	*N*	Response				
		Strongly agree	Agree	Neither agree nor disagree	Disagree	Strongly Disagree
*Candidate*						
My oral examination examiners were professional.	561	322 (57.4%)	200 (35.7%)	28 (5.0%)	10 (1.8%)	1 (0.2%)
My interactions with the examiners were smooth.	560	217 (38.8%)	254 (45.4%)	57 (10.2%)	28 (5.0%)	4 (0.7%)
I was able to demonstrate my knowledge and skills without obstacles.	561	146 (26.0%)	251 (44.7%)	88 (15.7%)	63 (11.2%)	13 (2.3%)
The ABA virtual oral examination effectively measures my ability to analyze clinical situations.	561	129 (23.0%)	246 (43.9%)	102 (18.2%)	59 (10.5%)	25 (4.5%)
The ABA virtual oral examination effectively measures my ability to adapt due to changing clinical scenarios.	559	135 (24.2%)	270 (48.3%)	84 (15.0%)	44 (7.9%)	26 (4.7%)
The ABA virtual oral examination effectively measures my ability to make appropriate clinical judgment for patient management.	561	140 (25.0%)	253 (45.1%)	97 (17.3%)	46 (8.2%)	25 (4.5%)
The ABA virtual oral examination effectively measures my ability to organize and present information.	561	145 (25.8%)	267(47.6%)	79 (14.1%)	49 (8.7%)	21 (3.7%)
**Which best describes your experience of the virtual SOE?**	796	**Positive**	**Neutral**	**Negative**		
		540 (67.8%)	224 (28.1%)	32 (4.0%)		
***Examiner***						
*(In comparison with the in-person SOE)*						
**How did the web-based nature of the ABA virtual oral examination affect your preparation effort?**	200	**Significantly more effort**	**Slightly more effort**	**Similar level of effort**	**Slightly less effort**	**Significantly less effort**
		14 (7.0%)	39 (19.5%)	130 (65.0%)	13 (6.5%)	4 (2.0%)
		**Significantly more difficult**	**Slightly more difficult**	**Similar level of difficulty**	**Slightly easier**	**Significantly easier**
**How would you rate the difficulty of the ABA virtual oral examination administration for examiners?**	201	12 (6.0%)	92 (45.8%)	75 (37.3%)	17 (8.5%)	5 (2.5%)
**How would you rate the difficulty of taking the ABA virtual oral examination for candidates?**	198	10 (5.1%)	66 (33.3%)	72 (36.4%)	46 (23.2%)	4 (2.0%)
**How would you rate the difficulty of the cases and guided questions in the ABA virtual oral examination?**	197	1 (0.5%)	11 (5.6%)	175 (88.8%)	10 (5.1%)	0 (0%)
		**Significantly better**	**Slightly better**	**Neither better nor worse**	**Slightly worse**	**Significantly worse**
**How was the overall flow of the ABA virtual oral examination?**	194	2 (1.0%)	12 (6.2%)	74 (38.1%)	98 (50.5%)	8 (4.1%)
**How were your interactions with your co-examiners in the ABA virtual oral examination?**	197	0 (0%)	4 (2.0%)	54 (27.4%)	85 (43.1%)	54 (27.4%)
**How were your interactions with candidates in the ABA virtual oral examination?**	197	0 (0%)	3 (1.5%)	105 (53.3%)	84 (42.6%)	5 (2.5%)
**How was candidate professionalism in the ABA virtual oral examination?**	195	**Significantly more professional**	**Slightly more professional**	**Similar level of professionalism**	**Slightly less professional**	**Significantly less professional**
		0 (0%)	0 (0%)	164 (84.1%)	30 (15.4%)	1 (0.5%)
**How did the web-based nature of the ABA virtual oral examination affect your ability to evaluate candidate performance?**	194	**Significantly more difficult to evaluate**	**Slightly more difficult to evaluate**	**Similar level of difficulty to evaluate**	**Slightly easier to evaluate**	**Significantly easier to evaluate**
		9 (4.6%)	66 (34.0%)	117 (60.3%)	2 (1.0%)	0 (0%)
**How would you rate the security of the ABA virtual oral examination?**	194	**Significantly more secure**	**Slightly more secure**	**Similar level of security**	**Slightly less secure**	**Significantly less secure**
		0 (0%)	2 (1.0%)	83 (42.8%)	77 (39.7%)	32 (16.5%)
**How was your development as an examiner in the ABA virtual oral examination?**	192	**Significantly more development**	**Slightly more development**	**Similar level of development**	**Slightly less development**	**Significantly less development**
		0 (0%)	9 (4.7%)	104 (54.2%)	60 (31.3%)	19 (9.9%)
**For an administration of 2 periods (i.e., 4 sessions) of the ABA oral examination, how was the level of fatigue associated with the ABA virtual oral examination administration?**	192	**Significantly more fatigue**	**Slightly more fatigue**	**Similar level of fatigue**	**Slightly less fatigue**	**Significantly less fatigue**
		25 (13.0%)	66 (34.4%)	79 (41.1%)	19 (9.9%)	3 (1.6%)
		**Strongly Agree**	**Agree**	**Neither Agree Nor Disagree**	**Disagree**	**Strongly Disagree**
The ABA virtual oral examination effectively measures candidates’ ability to analyze clinical situations.	192	94 (49.0%)	90 (46.9%)	7 (3.6%)	1 (0.5%)	0 (0%)
The ABA virtual oral examination effectively measures candidates’ ability to adapt due to changing clinical scenarios.	192	90 (46.9%)	88 (45.8%)	12 (6.3%)	2 (1.0%)	0 (0%)
The ABA virtual oral examination effectively measures candidates’ ability to make appropriate clinical judgment for patient management.	192	90 (46.9%)	93 (48.4%)	8 (4.2%)	1 (0.5%)	0 (0%)
The ABA virtual oral examination effectively measures candidates’ ability to organize and present information.	191	96 (50.3%)	84 (44.0%)	10 (5.2%)	1 (0.5%)	0 (0%)

Although cases and guided questions were of similar difficulty between the in-person and virtual SOEs, examiners tended to perceive that the virtual format was a more difficult experience for both candidates (38% considered virtual as being more difficult vs. 25% easier) and examiners (52% considered virtual more difficult vs. 11% easier). 45% of SOE examiners believed that their interactions with virtual candidates were not as effective as in-person and 39% found it more challenging to evaluate candidates virtually. In addition, 56%, 47%, and 41% of examiners cited less secure exam, “Zoom fatigue”, and less examiner development as additional negative factors of the virtual SOE, respectively (Table 4).

Of the 113 “repeaters” who took both the in-person and virtual SOEs, 28% reported that the web-based nature of the virtual SOE prompted them to increase their preparatory efforts (Supplemental Table 3). It’s unclear as to whether this increased effort was related to the virtual format of the exam or because they were re-attempting the exam, although 65% of this cohort reported a similar level of preparation as with their previous failed attempt. Despite their favorable view of the level of professionalism exhibited by their examiners, more candidates considered the virtual format to have hindered rather than helped their interaction with examiners. There was almost an even split in candidates’ perception of whether the web-based nature of the virtual SOE positively or negatively affected their ability to demonstrate their proficiency: 23% perceived a positive effect, 20% a negative effect, and 57% were neutral. This poses an interesting question as to whether the proximity and immediacy of an in-person examination is a necessary stressor to help identify candidates who will demonstrate the attributes of an ABA diplomate in pressurized clinical situations versus an artificial impediment to the demonstration of a candidate’s ability because of nervousness or performance anxiety [14, 15].

## Perceptions of the virtual OSCE

79% of virtual OSCE candidates indicated that the actors playing standardized patients or standardized clinicians portrayed the clinical scenarios authentically and 89% had “smooth” interactions with them (Table 5). These data are reassuring in the context of an exam requiring complex interactions with actors in multiple stations and quick task and role switching between stations. Other responses were less assuring — while 75% of OSCE candidates considered themselves to be able to demonstrate their knowledge and skills without obstacles, up to 30% of OSCE candidates were at best neutral regarding the virtual OSCE’s ability to effectively measure their communication skills and professionalism, and more than half of respondents were at best neutral that the virtual OSCE effectively measured their technical skills.

**Table 5:** Candidate and examiner experiences of the virtual Objective Structured Clinical Examination (OSCE)

	*N*	**Response**				
*Candidate*						
**How difficult was the OSCE portion of the APPLIED Exam for you?**	1267	**Very difficult**	**Somewhat difficult**	**Neither easy nor difficult**	**Not very difficult**	**Not difficult at all**
		48 (3.8%)	476 (37.6%)	479 (37.8%)	216 (17.0%)	48 (3.8%)
**How do you assess the relative difficulty of the two types of OSCE scenarios?**	1246	**Communication and professionalism scenarios were more difficult**	**Technical skills scenarios were more difficult**	**Both communication and professionalism scenarios and technical skills scenarios were equally difficult**		
		230 (18.5%)	614 (49.3%)	402 (32.3%)		
		**Strongly agree**	**Agree**	**Neither agree nor disagree**	**Disagree**	**Strongly Disagree**
The OSCE scenarios were relevant to skills I use in my practice.	1251	197 (15.7%)	588 (47.0%)	249 (19.9%)	160 (12.8%)	57 (4.6%)
The OSCE scenarios were sufficiently realistic.	1251	191 (15.3%)	616 (49.2%)	273 (21.8%)	124 (9.9%)	47 (3.8%)
*Fall candidates only for the following statements*						
The standardized patients or standardized clinicians portrayed the clinical scenarios authentically.	538	157 (29.2%)	270 (50.2%)	71 (13.2%)	32 (5.9%)	8 (1.5%)
My interactions with the standardized patients or standardized clinicians were smooth.	538	166 (30.9%)	312 (58.0%)	43 (8.0%)	13 (2.4%)	4 (0.7%)
I was able to demonstrate my knowledge and skills without obstacles.	537	119 (22.2%)	285 (53.1%)	81 (15.1%)	42 (7.8%)	10 (1.9%)
The ABA virtual OSCE effectively measures my communication skills.	537	120 (22.3%)	257 (47.9%)	106 (19.7%)	36 (6.7%)	18 (3.4%)
The ABA virtual OSCE effectively measures my professionalism.	538	128 (23.8%)	265 (49.3%)	93 (17.3%)	35 (6.5%)	17 (3.2%)
The ABA virtual OSCE effectively measures my technical skills.	537	68 (12.7%)	179 (33.3%)	147 (27.4%)	97 (18.1%)	46 (8.6%)
***Examiner***						
*(In comparison with the In-person OSCE)*						
**How did the web-based nature of the ABA virtual OSCE affect your preparation effort?**	170	**Significantly more effort**	**Slightly more effort**	**Similar level of effort**	**Slightly less effort**	**Significantly less effort**
		0 (0%)	16 (9.4%)	125 (73.5%)	21 (12.4%)	8 (4.7%)
		**Significantly more difficult**	**Slightly more difficult**	**Similar level of difficulty**	**Slightly easier**	**Significantly easier**
**How would you rate the difficulty of taking the ABA virtual OSCE for candidates?**	169	7 (4.1%)	62 (36.7%)	79 (46.7%)	17 (10.1%)	4 (2.4%)
**How would you rate the overall difficulty of the Communication and Professionalism stations in the ABA virtual OSCE?**	113	2 (1.8%)	15 (13.3%)	91 (80.5%)	5 (4.4%)	0 (0%)
**How would you rate the overall difficulty of the technical stations in the ABA virtual OSCE?**	65	0 (0%)	11 (16.9%)	52 (80.0%)	1 (1.5%)	1 (1.5%)
**How authentically did the standardized patients/standardized clinicians portray the clinical scenarios in the ABA virtual OSCE?**	113	**Significantly more authentically**	**Slightly more authentically**	**Similar level of authenticity**	**Slightly less authentically**	**Significantly less authentically**
		0 (0%)	3 (2.7%)	91 (80.5%)	19 (16.8%)	0 (0%)
**How did the web-based nature of the ABA virtual OSCE allow candidates to demonstrate their proficiency?**	163	**Significantly better**	**Slightly better**	**Neither better nor worse**	**Slightly worse**	**Significantly worse**
		0 (0%)	0 (0%)	120 (73.6%)	40 (24.5%)	3 (1.8%)
**How did the web-based nature of the ABA virtual OSCE affect your ability to evaluate candidate performance?**	163	**Significantly more difficult to evaluate**	**Slightly more difficult to evaluate**	**Similar level of difficulty to evaluate**	**Slightly easier to evaluate**	**Significantly easier to evaluate**
		3 (1.8%)	27 (16.6%)	123 (75.5%)	6 (3.7%)	4 (2.5%)
**How was the candidate professionalism in the ABA virtual OSCE?**	161	**Significantly more professional**	**Slightly more professional**	**Similar level of professionalism**	**Slightly less professional**	**Significantly less professional**
		0 (0%)	1 (0.6%)	147 (91.3%)	12 (7.5%)	1 (0.6%)
**How would you rate the security of the ABA virtual OSCE?**	162	**Significantly more secure**	**Slightly more secure**	**Similar level of security**	**Slightly less secure**	**Significantly less secure**
		1 (0.6%)	0 (0%)	97 (59.9%)	50 (30.9%)	14 (8.6%)
		**Strongly Agree**	**Agree**	**Neither Agree Nor Disagree**	**Disagree**	**Strongly Disagree**
The ABA virtual OSCE effectively measures candidates’ communication skills.	112	35 (31.3%)	64 (57.1%)	10 (8.9%)	3 (2.7%)	0 (0%)
The ABA virtual OSCE effectively measures candidates’ professionalism.	112	27 (24.1%)	64 (57.1%)	16 (14.3%)	5 (4.5%)	0 (0%)
The ABA virtual OSCE effectively measures candidates’ technical skills.	64	21 (32.8%)	30 (46.9%)	8 (12.5%)	5 (7.8%)	0 (0%)

Examiners perceived the virtual OSCE to be more difficult for the candidates than the in-person format (41% believed the virtual format to be more difficult vs. 12% easier). 26% of examiners considered the virtual OSCE to be worse than the in-person format for allowing candidates to demonstrate their proficiency, and the other 74% were neutral. Although a few examiners disagreed with statements that the virtual OSCE effectively measured candidate attributes (disagreement rates for technical skills, professionalism, and communication skills were 8%, 4%, and 3%, respectively), examiners overall had more faith than candidates in the virtual OSCE’s ability to effectively measure candidate attributes (Table 5). In comparison with the in-person OSCE, examiners were concerned about virtual OSCE’s security (40% less secure vs. < 1% more secure), the authenticity of actors portraying the clinical scenarios in the virtual format (17% less authentically vs. 3% more authentically), candidates’ ability to demonstrate their proficiency (26% worse vs. 0% better), and their ability to evaluate candidate performance (18% more difficult vs. 6% easier; Table 5).

Of the 28 virtual OSCE respondents who had previously taken the in-person OSCE, 15 (54%) reported more preparatory effort for the virtual OSCE than for their previous in-person OSCE. Although 89% of OSCE retakers thought the actors virtually portrayed the scenarios authentically, about 40% of them felt that the virtual OSCE made their interactions with the actors more difficult than when in person and negatively affected their ability to demonstrate proficiency (Supplemental Table 4). This recollection is consistent with our previous psychometric analysis showing that OSCE scenarios were more difficult when administered virtually compared with in-person [9]. 46% of OSCE retakers agreed that the virtual OSCE effectively measured their communication skills (vs. 39% who disagreed) and professionalism (vs.29% who disagreed). Of even more concern, only 25% agreed that the virtual OSCE effectively measured their technical skills (vs. 50% disagreed) and 59% considered the virtual technical stations more difficult than the in-person format (vs. 0% easier).

## Security

Security concerns associated with high-stakes virtual exams are well-recognized [16, 17]. The direct and real-time audiovisual interactions of the VAE mitigate some of the concerns associated with computer-based written examinations. Nonetheless, multiple possibilities for security breaches remain, including taking screenshots or video of exam scenarios, monitoring loops or echocardiogram images for later distribution, or surreptitiously receiving real-time aid from an unseen helper. At the assessment center, in-person candidates are prohibited from bringing electronic devices or other possible aids into the orientation or examination rooms. Such strict controls are not possible in the virtual format. The presumption was that candidates would adhere to the legally binding agreement they signed at examination registration and act honestly, honorably, and professionally. Some exam audiovisual recordings were reviewed by ABA Directors when concerns regarding irregular behavior or possible cheating were raised by examiners or staff. No cheating was detected, and no candidates had their VAE invalidated due to exam misconduct, although the absence of evidence of cheating does not necessarily mean evidence of absence. Of note, typically 2 to 3 candidates per year have their ABA *in-person written* exams invalidated because of breaches of the exam rules. Virtual exams pose inherent challenges in ensuring a fully secure environment — 56% of SOE examiners and 40% of OSCE examiners believed that the virtual SOE and virtual OSCE were less secure than their in-person equivalents, respectively. The remaining examiners thought that security was similar between the two formats (Tables 4 and 5). Security concerns did influence one major operational decision: while SOE guided questions and OSCE scenarios from in-person administrations remain active in the examination bank, the exam materials used virtually were discarded because of the potential for compromise. This considerably increases the cost and effort associated with generating new scenarios and questions for future examinations.

## Examiner mentorship

Examiners reported that training, mentorship, networking, and camaraderie suffered in the virtual format (Table 4), which was also reflected in many free-text comments not reported here. The significance should not be underestimated. An excellent examiner pool requires examiners’ sustained commitment to examine and continuous guidance and advice from examiner mentors. Networking opportunities are highly valued by examiners, and long-standing professional relationships and friendships are made and maintained over time. Even within a single year of remote examinations, it was clear that examiner interaction suffered by not being together on-site. 71% of the virtual SOE examiners reported that their interactions with fellow examiners were worse than when in-person and 41% believed that their development as an examiner was negatively affected because of the virtual format. These could have adverse implications for future exams. Other ABMS member boards have reported on similar sentiments, and the American Board of Emergency Medicine (personal communication, 2023) and the American Board of Obstetrics and Gynecology [18] changed – at least temporarily – to a hybrid exam model in which examiners are physically together in an examination center and candidates across the country are examined remotely.

## Return to in-person examinations

Determination of whether a physician possesses the attributes required to achieve certification by a medical specialty board is a high-stakes endeavor with implications for the physician, the specialty, and the public [19, 20]. The ABA successfully administered the VAE to its 3059 candidates in a single year, 2724 of whom became certified in anesthesiology, in a manner both practically feasible and psychometrically reliable [8, 9]. As vaccination against COVID-19 became widely available and pandemic-related disruptions lessened, the ABA, despite the successes of the VAE, returned to in-person testing for the APPLIED Examination in February 2022. Universal masking, the requirement of proof of vaccination for examiners and candidates (with some exceptions for candidates), COVID-19 contact tracing, and other infection prevention and mitigation measures were enforced. The planned six weeks of exams in 2022 were successfully completed for 2165 candidates, and similarly, 2117 candidates were examined in-person in 2023. Other certifying boards faced similar decisions about returning to in-person or staying virtual and made the same or different choices [18, 21–26].

The ABA’s return to the in-person format for the APPLIED Examination was based on several considerations with standardization and security foremost. The variability of internet speed, quality, and reliability across the country means that candidates have a less standardized experience than when the exam is taken in person. This inconsistency increases the potential for construct-irrelevant variance due to technical disruptions and technology-related candidate or examiner anxiety [27]. Despite taking steps to mitigate the risks, virtual exams are more open to breaches of security than those conducted in the tightly controlled in-person environment of the assessment center. There are additional concerns about the ability to remotely test communication and professionalism skills that are required to be used in person during daily practice, and the virtually-administered OSCE technical stations appear to be more difficult [28]. Current technology does not allow assessing some important content in a virtual format, such as physically acquiring and interpreting ultrasound images. Other potential domains for future assessment, such as team-based assessment and multi-disciplinary collaborative assessment, would also be difficult to conduct in a non-standard environment.

Development and implementation of the VAE were associated with substantial short-term investment, including the financial implications of reassignment of ABA personnel from other projects to work on the VAE, the costs of software development and licensing fees, and the need to hire temporary staff to act as exam facilitators. In keeping with reports from other ABMS member boards, [16, 18] the virtual examination was very labor-intensive for ABA staff. The ABA’s examiner-associated travel costs (flights, hotels, meals, etc.) were lower during the VAE. Weighed against this, however, was the clearly articulated diminished examiner experience, which raises concerns about volunteer examiner engagement, mentorship, and sustainability. Candidate expenses are lower for the virtual format - travel and hotel costs are negated and time away from work is likely to be less. The ABA is not unsympathetic to these considerations for early career anesthesiologists, but must weigh all factors holistically to ensure the fairness, integrity, and sustainability of the certification examination process.

## Experiences of other ABMS member boards

Until the introduction of an in-person OSCE by the American Board of Urology in 2023, the ABA was the only ABMS member board to utilize an OSCE. However, 14 ABMS member boards used some form of oral examination as a component of their initial certification process, and all converted to virtual delivery during the pandemic [20, 29]. Several ABMS member boards, including the American Boards of Emergency Medicine, Obstetrics and Gynecology, Ophthalmology, and Surgery, have published their experience of virtual oral exams, with varying levels of detail [18, 21, 23–25]. The nature of the virtual exams differed from their respective in-person exams for some boards (e.g., major changes in the duration of exam sessions and the number of examiners each candidate sees, abandonment of the use of images or videos). While the ABA managed the technology infrastructure in-house and directly hired temporary staff for exam proctoring/administration, some boards utilized vendors to manage those responsibilities. Post-pandemic plans also vary. Some boards have transitioned back to in-person exams, [30, 31] others continued the virtual format, [23, 32] and still others were exploring other formats such as bringing examiners physically together at an exam center to examine remote candidates [33].

## Lessons learned

The year-long administration of the ABA VAE demonstrated the capability of a medical specialty certifying board to remotely deliver high-stakes SOEs and OSCEs to thousands of physicians in a practically feasible and psychometrically rigorous manner. The technology required for internet-based examinations worked, albeit not perfectly and with concerns for lack of standardization. The urgent nature of its development meant that the VAE required a rapid conversion of well-established in-person examination procedures to a remote format; under less exigent circumstances, more innovative methods of remote assessment could be designed, pilot-tested, introduced, and refined over a longer timeline. Multiple methodologies to communicate with candidates and examiners are necessary. Minor practical details can have significant implications, especially where technology interfaces with humans under stressful, time-sensitive circumstances. Although cheating was neither expected nor detected, examination security remains a major concern. The VAE experience documented the cognitive and practical difficulties associated with remote exam delivery, highlighted the dedication of the ABA staff and a large cohort of volunteer anesthesiologist examiners, and reinforced the value of the in-person examiner experience. Finally, and perhaps most importantly, the VAE experience demonstrated the resilience of the early-career anesthesiologists who adapted to multiple changes and prepared for this major professional milestone despite their examinations, careers and lives being disrupted by the COVID-19 pandemic.

### Electronic supplementary material﻿

Below is the link to the electronic supplementary material.

Supplementary Material 1

## Data Availability

The datasets generated and/or analyzed during the current study are not publicly available due to the confidentiality and sensitivity of the survey data and the stated terms with the survey respondents, but they can be made available from the Corresponding Author on reasonable requests.
