# Supplementary material for: Administration of the American Board of Anesthesiology’s virtual APPLIED Examination: successes, challenges, and lessons learned
Source: BMC Med Educ. 2024 Jul 11;24:749. doi: 10.1186/s12909-024-05694-7 (PMC11241991; doi:10.1186/s12909-024-05694-7)
Supplement: Supplementary file 1 — Supplementary Material 1 [file 12909_2024_5694_MOESM1_ESM.docx]

**Supplemental Table 1**

Candidate survey results from the two-day December 2020 ABA Virtual APPLIED Examination pilot

|  | ***N*** | **Response** | | | | | | | | | | | |
| --- | --- | --- | --- | --- | --- | --- | --- | --- | --- | --- | --- | --- | --- |
| ***Support*** | | | | | | | | | | | | | |
| **How clearly did the ABA communicate what was required of you prior to the exam?** | 63 | **Extremely clearly** | | **Very clearly** | | **Somewhat clearly** | | | **Not so clearly** | | **Not at all clearly** | | |
|  |  | 33 (52.4%) | | 23 (36.5%) | | 5 (7.9%) | | | 1 (1.6%) | | 1 (1.6%) | | |
| **How timely were exam materials and information delivered to you?** | 62 | **Very timely. I received everything I needed well in advance** | | **On time. I had what I needed in time for the exam** | | **Late. I was left scrambling at the last minute** | | | | | | | |
|  |  | 21 (33.9%) | | 36 (58.1%) | | 5 (8.1%) | | | | | | | |
| **How useful did you find each of the exam materials?** |  | **Extremely useful** | | **Very useful** | | **Somewhat useful** | | | **Not so useful** | | **Not applicable** | | |
| Candidate Procedures Manual | 64 | 28 (43.8%) | | 28 (43.8%) | | 8 (12.5%) | | | 0 (0%) | | 0 (0%) | | |
| Live Webinar (held Dec. 9, 2020) | 64 | 14 (21.9%) | | 28 (43.8%) | | 13 (20.3%) | | | 1 (1.6%) | | 8 (12.5%) | | |
| Virtual Exam Infographic | 64 | 15 (23.4%) | | 28 (43.8%) | | 14 (21.9%) | | | 0 (0%) | | 7 (10.9%) | | |
| SOE Overview Video | 63 | 14 (22.2%) | | 24 (38.1%) | | 17 (27.0%) | | | 1 (1.6%) | | 7 (11.1%) | | |
| OSCE Overview Video | 63 | 12 (19.0%) | | 26 (41.3%) | | 18 (28.6%) | | | 1 (1.6%) | | 6 (9.5%) | | |
| **How did you prepare for the OSCE?** | 62 | **Practice scenarios in residency/fellowship program** | | **Review books** | | **Review courses** | | | **Online tutorials** | | **Other** | | **No preparation** |
|  |  | 29 (46.8%) | | 5 (8.1%) | | 6 (9.7%) | | | 10 (16.1%) | | 7 (11.3%) | | 5 (8.1%) |
| **How helpful were the OSCE scenario videos on the website in helping you prepare for the OSCE?** | 62 | **Very helpful** | | **Somewhat helpful** | | **Not very helpful** | | | **Not at all helpful** | | **Did not review OSCE scenario videos** | | |
|  |  | 32 (51.6%) | | 19 (30.6%) | | 3 (4.8%) | | | 0 (0%) | | 8 (12.9%) | | |
| **As you first entered the virtual exam, were exam administrators helpful to your experience?** | 64 | **Extremely helpful** | | **Very helpful** | | **Somewhat helpful** | | | **Not so helpful** | | **Not at all helpful** | | |
|  |  | 27 (42.2%) | | 33 (51.6%) | | 4 (6.3%) | | | 0 (0%) | | 0 (0%) | | |
| ***Technical*** | | | | | | | | | | | | | |
| **Which best describes your experience with the system check?** | 64 | **My system passed on the first try** | | **I had to make a few adjustments to my system before it passed** | | **I had to make substantial adjustments to my system before it passed** | | | **I had to choose a different location and/or network for the exam** | | **Other** | | |
|  |  | 61 (95.3%) | | 2 (3.1%) | | 0 (0%) | | | 0 (0%) | | 1 (1.6%) | | |
| **How often did your exams start at the scheduled time?** | 64 | **Always** | | **Often** | | **Sometimes** | | | **Never** | | | | |
|  |  | 34 (53.1%) | | 18 (28.1%) | | 8 (12.5%) | | | 4 (6.3%) | | | | |
| **How often did you lose connectivity during the exam?** | 64 | **Always** | | **Often** | | **Sometimes** | | | **Never** | | | | |
|  |  | 0 (0%) | | 0 (0%) | | 15 (23.4%) | | | 49 (76.6%) | | | | |
| **Which of the following best describes your experience with the Zoom platform?** | 64 | **Exceeded expectations** | | **Met expectations** | | **Below expectations** | | | | | | | |
|  |  | 18 (28.1%) | | 45 (70.3%) | | 1 (1.6%) | | | | | | | |
| **What best describes your experience of each of the following as it pertains to both sessions of your Standardized Oral Exam (SOE)?** |  | **No issues** | | **A few issues** | | **Significant issues** | | | **N/A** | | | | |
| Zoom: Audio | 62 | 46 (74.2%) | | 14 (22.6%) | | 2 (3.2%) | | | 0 (0%) | | | | |
| Zoom: Video | 62 | 53 (85.5%) | | 7 (11.3%) | | 2 (3.2%) | | | 0 (0%) | | | | |
| Zoom: Connectivity | 60 | 48 (80.0%) | | 10 (16.7%) | | 2 (3.3%) | | | 0 (0%) | | | | |
| Stem: Display | 61 | 43 (70.5%) | | 14 (23.0%) | | 4 (6.6%) | | | 0 (0%) | | | | |
| Stem: Prep time | 62 | 49 (79.0%) | | 10 (16.1%) | | 3 (4.8%) | | | 0 (0%) | | | | |
| Stem: Demonstration time | 62 | 56 (90.3%) | | 4 (6.5%) | | 2 (3.2%) | | | 0 (0%) | | | | |
| **What best describes your experience of each of the following as it pertains to all 7 scenarios of your Objective Structured Clinical Exam (OSCE)?** |  | **No issues** | | **A few issues** | | **Significant issues** | | | **N/A** | | | | |
| Zoom: Audio | 62 | 56 (90.3%) | | 5 (8.1%) | | 0 (0%) | | | 1 (1.6%) | | | | |
| Zoom: Video | 62 | 56 (90.3%) | | 5 (8.1%) | | 0 (0%) | | | 1 (1.6%) | | | | |
| Zoom: Connectivity | 62 | 55 (88.7%) | | 6 (9.7%) | | 0 (0%) | | | 1 (1.6%) | | | | |
| Stem: Display | 62 | 45 (72.6%) | | 15 (24.2%) | | 1 (1.6%) | | | 1 (1.6%) | | | | |
| Stem: Prep time | 61 | 45 (73.8%) | | 10 (16.4%) | | 5 (8.2%) | | | 1 (1.6%) | | | | |
| Standardized Patient Experience | 62 | 56 (90.3%) | | 4 (6.5%) | | 1 (1.6%) | | | 1 (1.6%) | | | | |
| Technical Stations | 62 | 47 (75.8%) | | 13 (21.0%) | | 2 (3.2%) | | | 0 (0%) | | | | |
| **Were you aware of any technical issues on the part of your examiners?** | 64 | **Yes** | | **No** | | | | | | | | | |
|  |  | 15 (23.4%) | | 49 (76.6%) | | | | | | | | | |
| ***Content & Validity*** | | | | | | | | | | | | | |
| **How difficult was the OSCE portion of the APPLIED Exam for you?** | 61 | **Very difficult** | | **Somewhat difficult** | | **Neither easy nor difficult** | | | **Not very difficult** | | **Not difficult at all** | | |
|  |  | 0 (0%) | | 23 (37.7%) | | 25 (41.0%) | | | 10 (16.4%) | | 3 (4.9%) | | |
| **How do you assess the relative difficulty of the two types of OSCE scenarios?** | 61 | **Communication & professionalism scenarios were more difficult** | | **Technical skills scenarios were more difficult** | | **The two types of OSCE scenarios were equally difficult** | | | | | | | |
|  |  | 12 (19.7%) | | 31 (50.8%) | | 18 (29.5%) | | | | | | | |
| **The OSCE scenarios were relevant to skills I use in my practice.** | 61 | **Strongly agree** | | **Agree** | | **Neither agree nor disagree** | | | **Disagree** | | **Strongly Disagree** | | |
|  |  | 11 (18.0%) | | 32 (52.5%) | | 10 (16.4%) | | | 6 (9.8%) | | 2 (3.3%) | | |
| **The OSCE scenarios were sufficiently realistic.** | 61 | **Strongly agree** | | **Agree** | | **Neither agree nor disagree** | | | **Disagree** | | **Strongly Disagree** | | |
|  |  | 14 (23.0%) | | 33 (54.1%) | | 5 (8.2%) | | | 8 (13.1%) | | 1 (1.6%) | | |
| ***Overall*** | | | | | | | | | | | | | |
| **Which best describes your experience of the virtual SOE?** | 62 | **Positive** | **Neutral** | | **Negative** | | | | | | | | |
|  |  | 49 (79.0%) | 12 (19.4%) | | 1 (1.6%) | | | | | | | | |
| **How would you rate the overall experience of your virtual APPLIED Exam?** | 70 | **Very satisfied** | **Satisfied** | | **Somewhat**  **satisfied** | | **Neither satisfied nor dissatisfied** | **Somewhat dissatisfied** | | **Dissatisfied** | | **Very dissatisfied** | |
|  |  | 19 (27.1%) | 30 (42.9%) | | 16 (22.9%) | | 2 (2.9%) | 2 (2.9%) | | 1 (1.4%) | | 0 (0%) | |

**Supplemental Table 2**

Examiner survey data from the two-day December 2020 ABA Virtual APPLIED Examination pilot

|  | ***N*** | **Response** | | | | |
| --- | --- | --- | --- | --- | --- | --- |
| ***Support*** | | | | | | |
| **How clearly did the ABA communicate what was required of you prior to the exam?** | 26 | **Extremely clearly** | **Very clearly** | **Somewhat clearly** | **Not so clearly** | **Not at all clearly** |
|  |  | 7 (26.9%) | 11 (42.3%) | 4 (15.4%) | 4 (15.4%) | 0 (0%) |
| **How timely were exam materials and information delivered to you?** | 26 | **Very timely. I received everything I needed well in advance.** | **On time. I had what I needed in time for the exam.** | **Late. I was left scrambling at the last minute.** | **Other** | |
|  |  | 13 (50.0%) | 12 (46.2%) | 1 (3.8%) | 0 (0%) | |
| ***Technical*** | | | | | | |
| **Which best describes your experience with the system check?** | 26 | **My system passed on the first try** | **I had to make a few adjustments to my system before it passed** | **I had to make substantial adjustments to my system before it passed** | **I had to choose a different location and/or network for the exam** | **Other** |
|  |  | 21 (80.8%) | 4 (15.4%) | 0 (0%) | 0 (0%) | 1 (3.8%) |
| **Which of the following best describes your experience with the Zoom platform?** | 23 | **Exceeded expectations** | **Met expectations** | **Below expectations** | | |
|  |  | 3 (13.0%) | 19 (82.6%) | 1 (4.3%) | | |
| **How often did your exams start at the scheduled time?** | 22 | **Always** | **Often** | **Sometimes** | **Never** | |
|  |  | 8 (36.4%) | 11 (50.0%) | 3 (13.6%) | 0 (0%) | |
| **How often did you lose connectivity during the exam?** | 23 | **Always** | **Often** | **Sometimes** | **Never** | |
|  |  | 0 (0%) | 0 (0%) | 4 (17.4%) | 19 (82.6%) | |
| ***Overall*** | | | | | | |
| **How would you describe your SOE examination/auditing experience?** | 20 | **Very positive** | **Positive** | **Neutral** | **Negative** | |
|  |  | 2 (10.0%) | 13 (65.0%) | 5 (25.0%) | 0 (0%) | |
| **How would you rate your experience of the virtual APPLIED Exam?** | 26 | **Excellent** | **Good** | **Fair** | | |
|  |  | 0 (0%) | 26 (100%) | 0 (0%) | | |

**Supplemental Table 3**

Repeat candidate perception of the virtual Standardized Oral Examination (SOE)

|  | ***N*** | **Response** | | | | |
| --- | --- | --- | --- | --- | --- | --- |
| ***(****In comparison with the in-person SOE)* | | | | | | |
| **How did the web-based nature of the ABA virtual oral examination affect your preparation effort?** | 113 | **Significantly more effort** | **Slightly more effort** | **Similar level of effort** | **Slightly less effort** | **Significantly less effort** |
|  |  | 15 (13.3%) | 17 (15.0%) | 73 (64.6%) | 4 (3.5%) | 4 (3.5%) |
| **How was the level of examiner professionalism different in the ABA virtual oral examination?** | 113 | **Significantly more professional** | **Slightly more professional** | **Similar level of professionalism** | **Slightly less professional** | **Significantly less professional** |
|  |  | 5 (4.4%) | 12 (10.6%) | 94 (83.2%) | 2 (1.8%) | 0 (0%) |
| **How did the web-based nature of the ABA virtual oral examination affect your interaction with examiners?** | 112 | **Significantly more difficult** | **Slightly more difficult** | **Neither more difficult nor easier** | **Slightly easier** | **Significantly easier** |
|  |  | 5 (4.5%) | 35 (31.3%) | 50 (44.6%) | 15 (13.4%) | 7 (6.3%) |
| **How did the web-based nature of the ABA virtual oral examination affect your ability to demonstrate your proficiency?** | 113 | **Significantly better** | **Slightly better** | **Neither better nor worse** | **Slightly worse** | **Significantly worse** |
|  |  | 10 (8.8%) | 16 (14.2%) | 64 (56.6%) | 20 (17.7%) | 3 (2.7%) |
|  | | | | | | |
|  |  | **Strongly agree** | **Agree** | **Neither agree nor disagree** | **Disagree** | **Strongly disagree** |
| The ABA virtual oral examination effectively measures my ability to analyze clinical situations. | 113 | 34 (30.1%) | 45 (39.8%) | 21 (18.6%) | 9 (8.0%) | 4 (3.5%) |
| The ABA virtual oral examination effectively measures my ability to adapt due to changing clinical scenarios. | 112 | 29 (25.9%) | 49 (43.8%) | 19 (17.0%) | 10 (8.9%) | 5 (4.5%) |
| The ABA virtual oral examination effectively measures my ability to make appropriate clinical judgment for patient management. | 112 | 31 (27.7%) | 48 (42.9%) | 22 (19.6%) | 7 (6.3%) | 4 (3.6%) |
| The ABA virtual oral examination effectively measures my ability to organize and present information. | 112 | 30 (26.8%) | 51 (45.5%) | 20 (17.9%) | 8 (7.1%) | 3 (2.7%) |

**Supplemental Table 4**

Repeat candidate perception of the virtual Objective Structured Clinical Examination (OSCE)

|  | ***N*** | **Response** | | | | |
| --- | --- | --- | --- | --- | --- | --- |
| ***(****In comparison with the in-person OSCE)* | | | | | | |
| **How did the web-based nature of the ABA virtual OSCE affect your preparation effort?** | 28 | **Significantly more effort** | **Slightly more effort** | **Similar level of effort** | **Slightly less effort** | **Significantly less effort** |
|  |  | 6 (21.4%) | 9 (32.1%) | 13 (46.4%) | 0 (0%) | 0 (0%) |
| **How authentically did standardized patients or standardized clinicians portray the clinical scenarios in the ABA virtual OSCE?** | 28 | **Significantly more authentically** | **Slightly more authentically** | **Similar level of authenticity** | **Slightly less authentically** | **Significantly less authentically** |
|  |  | 0 (0%) | 2 (7.1%) | 23 (82.1%) | 2 (7.1%) | 1 (3.6%) |
| **How did the web-based nature of the ABA virtual OSCE affect your interaction with standardized patients or standardized clinicians?** | 28 | **Significantly more difficult** | **Slightly more difficult** | **Neither more difficult nor easier** | **Slightly easier** | **Significantly easier** |
|  |  | 4 (14.3%) | 8 (28.6%) | 15 (53.6%) | 0 (0%) | 1 (3.6%) |
| **How did the web-based nature of the ABA virtual OSCE affect your ability to demonstrate your proficiency?** | 28 | **Significantly better** | **Slightly better** | **Neither better nor worse** | **Slightly worse** | **Significantly worse** |
|  |  | 1 (3.6%) | 0 (0%) | 16 (57.1%) | 8 (28.6%) | 3 (10.7%) |
| **How was the difficulty level of the technical stations (interpretation of monitors, interpretation of echocardiograms)?** | 27 | **Significantly more difficult** | **Slightly more difficult** | **Similar level of difficulty** | **Slightly easier** | **Significantly easier** |
|  |  | 5 (18.5%) | 11 (40.7%) | 11 (40.7%) | 0 (0%) | 0 (0%) |
|  | | | | | | |
|  |  | **Strongly agree** | **Agree** | **Neither agree nor disagree** | **Disagree** | **Strongly disagree** |
| The ABA virtual OSCE effectively measures my communication skills. | 28 | 4 (14.3%) | 9 (32.1%) | 4 (14.3%) | 8 (28.6%) | 3 (10.7%) |
| The ABA virtual OSCE effectively measures my professionalism. | 28 | 3 (10.7%) | 10 (35.7%) | 7 (25.0%) | 6 (21.4%) | 2 (7.1%) |
| The ABA virtual OSCE effectively measures my technical skills. | 28 | 2 (7.1%) | 5 (17.9%) | 7 (25.0%) | 11 (39.3%) | 3 (10.7%) |
